# Genetic susceptibility to Chagas disease cardiomyopathy: involvement of several genes of the innate immunity and chemokine-dependent migration pathways

**DOI:** 10.1186/1471-2334-13-587

**Published:** 2013-12-12

**Authors:** Amanda Farage Frade, Cristina Wide Pissetti, Barbara Maria Ianni, Bruno Saba, Hui Tzu Lin-Wang, Luciana Gabriel Nogueira, Ariana de Melo Borges, Paula Buck, Fabrício Dias, Monique Baron, Ludmila Rodrigues Pinto Ferreira, Andre Schmidt, José Antonio Marin-Neto, Mario Hirata, Marcelo Sampaio, Abílio Fragata, Alexandre Costa Pereira, Eduardo Donadi, Jorge Kalil, Virmondes Rodrigues, Edecio Cunha-Neto, Christophe Chevillard

**Affiliations:** 1 Heart Institute (InCor), University of São Paulo School of Medicine (FMUSP), Av. Dr. Enéas de Carvalho Aguiar, 44 Bloco 2 9º andar, São Paulo, SP 06504-000, Brazil; 2 Institute for Investigation in Immunology (iii), INCT, São Paulo, SP, Brazil; 3 Laboratory of Immunology, Universidade Federal do Triângulo Mineiro (UFTM), 40 Frei Paulino, Uberaba, MG 48036-180, Brazil; 4 Instituto de Cardiologia Dante Pazzanese (IDPC), Avenida Dante Pazzanese 500 - Ibirapuera, Sâo Paulo, SP 04012-909, Brazil; 5 School of Medicine of Ribeirão Preto (FMRP), University of São Paulo, Av. Bandeirantes, 4900 - Monte Alegre 15059-900, Ribeirão Preto, SP, Brazil; 6 Division of Clinical Immunology and Allergy, University of São Paulo School of Medicine, São Paulo, SP 06504-000, Brazil; 7 Aix-Marseille Université, INSERM, GIMP UMR_S906, Faculté de médecine, 27 bd Jean Moulin, Marseille, cedex 05 13385, France

**Keywords:** Chagas disease, Susceptibility, CCR5, CCL2, TIRAP

## Abstract

**Background:**

Chagas disease, caused by the protozoan *Trypanosoma cruzi* is endemic in Latin America. Thirty percent of infected individuals develop chronic Chagas cardiomyopathy (CCC), an inflammatory dilated cardiomyopathy that is, by far, the most important clinical consequence of *T. cruzi* infection. The others remain asymptomatic (ASY). A possible genetic component to disease progression was suggested by familial aggregation of cases and the association of markers of innate and adaptive immunity genes with CCC development. Migration of Th1-type T cells play a major role in myocardial damage.

**Methods:**

Our genetic analysis focused on CCR5, CCL2 and MAL/TIRAP genes. We used the Tag SNPs based approach, defined to catch all the genetic information from each gene. The study was conducted on a large Brazilian population including 315 CCC cases and 118 ASY subjects.

**Results:**

The CCL2rs2530797A/A and TIRAPrs8177376A/A were associated to an increase susceptibility whereas the CCR5rs3176763C/C genotype is associated to protection to CCC. These associations were confirmed when we restricted the analysis to severe CCC, characterized by a left ventricular ejection fraction under 40%.

**Conclusions:**

Our data show that polymorphisms affecting key molecules involved in several immune parameters (innate immunity signal transduction and T cell/monocyte migration) play a role in genetic susceptibility to CCC development. This also points out to the multigenic character of CCC, each polymorphism imparting a small contribution. The identification of genetic markers for CCC will provide information for pathogenesis as well as therapeutic targets.

## Background

Chagas disease (American trypanosomiasis) is caused by the protozoan *Trypanosoma cruzi* and transmitted by the reduviid bug. It occurs exclusively in the Americas, particularly in poor, rural areas of Mexico, Central America, and South America. The disease remains endemic in Latine America where the vector-based transmission is still active in some countries. Imported disease is increasingly recognized as an emerging problem in the USA and Europe due to immigration from Latin America. It is estimated that as many as 8–9 million people have Chagas disease. Approximately, 40 million people are currently at risk of infection [1]. Decades after acute infection, approximately 30% of infected individuals develop Chronic Chagas cardiomyopathy (CCC), one of the most important consequence of *T. cruzi* infection. CCC is an inflammatory dilated cardiomyopathy, with a potentially fatal outcome. 5 to 10% of infected individuals develop digestive disease. The remaining two-thirds of infected individuals remain asymptomatic (ASY) and free from heart disorders for life [2]. 20,000 deaths attributable to Chagas disease occur annually, typically due to CCC [3]. Heart failure due to CCC has a worse prognosis with 50% shorter survival when compared to other cardiomyopathies of different etiologies [4,5].

The dynamics of the immune response to *T. cruzi* is that of a persistent infection with an obligatory intracellular parasite. During acute *T. cruzi* infection, *T. cruzi* pathogen-associated molecular patterns (PAMPs) trigger innate immunity in multiple cell types [6], which release proinflammatory cytokines and chemokines, such as IL-1, IL-6, IL-12, IL-18, TNF-α, CCL2, CCL5, and CXCL9 activating and mobilizing migration of cascades of inflammatory cells [7,8]. Antigen-presenting cells subsequently elicit a strong T cell and antibody response against *T. cruzi,* where IL-12 and IL-18 drive the differentiation of IFN-γ-producing *T. cruzi*–specific Th1 T cells which migrate to sites of *T. cruzi*-induced inflammation, including the myocardium, in response to locally produced chemokines [9,10]. Th1 T cell and antibody responses lead to control but not complete elimination of tissue and blood parasitism, establishing a low-grade chronic persistent infection by *T. cruzi*. As a result of persistent infection, both CCC and ASY chronic Chagas disease patients show a skewed Th1-type immune response [11,12], but those who develop Chagas cardiomyopathy display a particularly strong Th1-type immune response with increased numbers of IFN-γ-producing T cells in peripheral blood mononuclear cells (PBMC) [13] as well as plasma TNF-α in comparison with uninfected or ASY patients [14]. PBMC of CCC patients also display increased levels of IFN-γ- or TNF-α producing CCR5/CXCR3+ CD4+ T cells [15,16]. In addition, CCC patients display a reduced number of CD4^+^CD25^high^IL-10^+^ and CD4^+^CD25^high^FoxP3^+^ regulatory T cells in their peripheral blood as compared to patients in the ASY form of Chagas disease, suggesting such cells may play a role in the control of the intensity of inflammation in chronic Chagas disease [15,17]. Furthermore, PBMC from CCC patients displayed increased numbers of CD4^+^CD25^high^FoxP3^+^CTLA-4^+^ T cells, and decreased numbers of as compared to ASY patients. These reports suggest that a smaller CD4^+^FoxP3+/CD25^+^ Treg compartment with deficient suppressive activity exists in CCC patients, leading to uncontrolled production of Th1 cytokines [18]. Circulating CD4^+^IL-17^+^ T cells appear in low frequency in PBMC from CCC patients as compared with ASY patients and non-infected individuals [18,19]. On the whole, these results suggest that proinflammatory cells and cytokines are markers associated with progression to CCC, whereas the production of IL-10, IL-17 and increased numbers of regulatory T cells are markers of protection from CCC development, indicating that failure to regulate Th1 responses may be the underlying immune defect of patients who progress to CCC.

The exacerbated Th1 response observed in the PBMC of CCC patients is reflected on the Th1-rich myocardial inflammatory infiltrate, with mononuclear cells predominantly producing IFN-γ and TNF-α, with lower production of IL-4, IL-6, IL-7, and IL-15 [7,20,21]. It has recently been shown that CCL5+, CCXCL9+, CCR5+, CXCR3+ cells were abundant in CCC myocardium, and mRNA levels of the Th1-chemoattracting chemokines CXCL9, CXCL10, CCL2 (also known as MCP-1), CCL3, CCL4, CCL5; along with CCL17, CCL19, CCL21 and their receptors were also found to be upregulated in CCC heart tissue [12,22]. Importantly, median expression of CCL5, a CCR5 ligand, was the highest among all chemokines tested (166-fold increase over control). Significantly, the intensity of the myocardial infiltrate was positively correlated with CXCL9 mRNA expression. Moreover, a single nucleotide polymorphism in the *CXCL9* gene, associated with a reduced risk of developing severe CCC in a cohort study, was associated with reduced CXCL9 expression and intensity of myocarditis in CCC [22]. These results are consistent with a major role of locally produced Th1-chemoattractant chemokines in the accumulation of CXCR3/CCR5+ Th1 T cells in CCC heart tissue [23].

Familial aggregation of CCC has been described, suggesting that there might be a genetic component to disease susceptibility [24]. Several genes were associated to an increased risk to develop cardiomyopathy (HLA, MHC, TNF, IL1A, IL1B, IL1RN, IL10, IL12B, TIRAP, CCL2, BAT1, LTA, IKBL, CCR5, MIF, IFNG, CXCL9, CXCL10) [25-50]. So far, up to 30 case control studies were done (see for review [51-53]). These studies often led to inconclusive results that may be explained in different ways: a) the use of seronegative subjects as controls which are inadequate controls, since it is unknown whether they were exposed to the pathogen; b) the relatively small size of the study groups which affected the power (the probability) to detect an association; c) the number of tested SNPs; d) the highly heterogeneous genetic background of the study population due to admixture; e) the sex ratio known to exist has not been taken in consideration [54].

Among these susceptibility studies, putative implication of genes crucially involved in the innate immunity-such as the Toll like receptors (TLR) and some of its most relevant signalling molecules like TIRAP was searched for. Two studies on the TLR and TIRAP failed to identify disease associations with TLR 1,2, 5, 6 and 9; in one of the reports an association was found with a TLR4 SNP among Chilean chagasic patients [55], while in the second study – which enrolled nearly double the number of Brazilian Chagasic individuals - no association was found with TLR4, but instead with TIRAP S180L heterozygosity [41]. Chemokines are key players in controlling migration of specific cell types bearing their receptors to sites of tissue inflammation, and associations between CCR5 –involved in T cell and macrophage migration and CCL2 –involved in monocyte migration - with CCC were reported [42,47,48]. Both processes, TLR signaling and chemokine-mediated cell migration are of paramount importance in Chagas disease and are key to the pathogenesis of CCC. Here, we conducted a study focusing on TIRAP, CCL2 and CCL5. Thorough genetic analysis, testing multiple tag SNPs per gene and thus detecting any possible relevant genetic variants in a large Brazilian population and ASY subjects as controls we could have a sensitive assessment of the contribution of genetic variants in prognosis to CCC either confirming or finding additional associated SNPs in the mentioned genes. This can be considered a candidate gene replication study, performed with a larger cohort of Chagas patients and only comparing CCC to the asymptomatic seropositive (ASY) patient group. Significant associations were found for CCR5, CCL2, and TIRAP genes.

## Methods

### Ethical standard

Written informed consent was obtained from all the patients, in accordance with the guidelines of the various internal review boards of all the involved institutions. The protocol was also approved by the INSERM Internal Review Board and the Brazilian National Ethics in Research Commission (CONEP). All the patients enrolled in this study were over 21 years old so paternal consent was not required. In the case of samples from heart donors, written informed consent was obtained from their families. Investigations were conformed to the principles outlined in the declaration of Helsinki.

### Diagnostic criteria

The diagnostic criteria for Chagas disease included the detection of antibodies against *T. cruzi* in at least two of three independent serological tests (EIA [Hemobio Chagas; Embrabio São Paulo], indirect immunofluorescence assays [IFA-immunocruzi; Biolab Merieux], and indirect hemagglutination tests [Biolab Merieux]) [12]. All Chagas disease patients underwent standard electrocardiography and echocardiography. Echocardiography was performed at the hospital, with a Sequoia model 512 echocardiograph with a broad-band transducer. Left ventricular dimensions and regional and global function, including the recording of left ventricular ejection fraction (LVEF), were evaluated with a two-dimensional, M-mode approach, in accordance with the recommendations of the American Society of Echocardiography. ASY subjects had no electrocardiography and echocardiography changes. CCC patients presented typical conduction abnormalities (right bundle branch block and/or left anterior division hemiblock) [56]. CCC patients with significant left ventricular systolic dysfunction (LVEF <40%) were classified as having severe CCC, whereas those with no significant ventricular dysfunction (LVEF ≥40%) were classified as having moderate CCC. We selected 40% as arbitrary cutoff value that has been previously used to define significant ventricular dysfunction by our group and others [22,57,58].

### Study population for polymorphism analysis

The patients and ASY controls were born and raised in rural areas of Sao Paulo, Minas Gerais and Bahia states and enrolled in one of the study centers (Incor, FMUSP, FMRP, UFTM, IDPC). Patients with digestive forms were excluded of this study. Patients were classified as ASY (*n* = 118) or as having CCC (*n* = 315). ASY individuals were used as the control subjects for this study because they were from the same areas of endemicity as the patients with CCC, had encountered the parasite and had tested seropositive for *T. cruzi* infection, but the infection had not progressed to CCC. Of the 118 ASY subjects, 45.3% were male, whereas in the CCC patients group, this percentage reaches 61.3%. The difference in sex distribution between the groups was significant (*p* = 1.21E-4; OR = 2.126; 95% CI: 1.450 – 3.12). It is well known that male patients infected with *T. cruzi* have a higher risk of progression to CCC than female patients [54,59,60]. Of 315 patients with CCC, 106 (42 men [39.6%] and 64 women [60.4%]) showed no significant ventricular dysfunction and were thus classified as having moderate CCC, whereas 199 (144 men [72.4%] and 55 women [27.6%]) had severe ventricular dysfunction and were classified as having severe CCC. Data for left ventricular ejection fraction were missing for 10 patients with CCC. So, when we compared moderate patients to severe patients, these 10 individuals were excluded from the analysis. Regarding progression of the ASY cases to CCC, the yearly progression rate –regardless of age group- is ca. 1-2%/year. The average age of Subjects with asymptomatic form was above 55 years. Taking into account that they were all born in endemic areas before vector transmission was interrupted, it is likely that in most if not all cases vector-borne infection occurred in early childhood. The odds that a significant number of such mature patients convert to CCC, and that this thwarts our statistical calculation is rather low; however, this is a pitfall of all cross sectional studies on diseases that display progression.

### Blood samples and DNA preparation

For each subject, 5 to 15 ml of blood were collected in EDTA tubes. Genomic DNA was isolated on a silica-membrane according to the manufacturer’s protocol (QIAamp DNA Blood Max Kit, Qiagen, Hilden, Germany).

### SNP selection

Tag single nucleotide polymorphisms (SNPs) were selected on the basis of HapMap Data for the Caucasian and Yoruba reference populations. Tag SNPs were selected within a region extending 5 kb on either side of the candidate gene. The minor allele frequency (MAF) cut off value was arbitrarily set at 20% (so the markers characterized by a MAF < 20% were excluded from the analysis by lack of power). In each reference population, the markers with MAF > 20% are included in different blocks of correlation (based on the r^2^ values). One marker in each block was selected and considered as a Tag SNPs. Indeed, markers located in the same block of correlation gave the same genetic information in association studies. Tag SNPs characterised by a MAF over 20% on at least one reference population were selected. These Tag SNPs were defined to catch all the genetic information from the candidate gene. We selected three tag SNPs for CCR5, six tag SNPs for CCL2 and six tag SNPs for MAL/TIRAP genes. Taking into account a disease with a prevalence of 30%, a cutoff for significant association of 0.05, for a genotype relative risk of 1.3, the probability to detect a real association reaches 63% with 315 chronic cases and 118 ASY controls. We decided to use a cut off of 20% instead of 10% or 15%. For lower cut off, the number of Tag SNPs will increase and it will request a seriously large your study population to have a good statistical power.

### SNP genotyping

Most of the genotyping was done with the Golden Gate genotyping assay (Illumina, San Diego, USA). In some cases, genotyping assays were performed with the *Taq*Man system (Applied Biosystems, Foster City, USA) according to the manufacturer’s instructions.

### Statistical analysis

SPSS Statistics software v. 17.0 (IBM, Armonk, USA) was used for statistical analyses. We performed stepwise binary logistic regression analysis on the whole population, to analyse the relationship between the probability of an individual to develop chronic Chagas cardiomyopathy and the main covariates (sex and polymorphisms). Sex was considered as a binary covariate. In our stepwise binary logistic regression analysis, genotypes were considered as binary covariates. Indeed, for each polymorphism we had two alleles (A frequent one; a rare one). So, we obtained three genotypes (AA, Aa and aa). In our stepwise binary logistic regression analysis, genotypes were considered as binary covariates. So, we performed three different analyses (Analysis 1: AA vs Aa + aa (we supposed that the a allele is dominant); Analysis 2: AA + aa vs Aa (we supposed that the heterozygote carriers are different from the homozygote ones); Analysis 3: AA + Aa vs aa (we supposed that the A allele is dominant)). The best results are indicated in Tables 1, 2 and 3.

**Table 1:** Association studies between CCC and ASY including as covariates the gender and the polymorphism one by one

**GENE**	**Tag SNP**	**Genotype groups**	**Association test**
**CCR5**	**rs3176763**	CC vs CA + AA	p = 0.006; OR = 1.79; 95% CI: 1.18-2.70
	**rs3087253**	AA vs AT + TT	p = 0.640; OR = 1.06; 95% CI: 0.84-1.32
	**rs11575815**	AA + AT vs TT	p = 0.030; OR = 1.41; 95% CI: 1.03-1.92
**CCL2**	**rs3760396**	GG vs GA + AA	p = 0.373; OR = 1.13; 95% CI: 0.87-1.46
	**rs2857656**	CC vs CG + GG	p = 0.440; OR = 1.09; 95% CI: 0.87-1.36
	**rs4586**	TT vs TC + CC	p = 0.032; OR = 1.30; 95% CI: 1.02-1.65
	**rs3917891**	CC vs CT + TT	p = 0.037; OR = 1.56; 95% CI: 1.03-2.37
	**rs2530797**	AA vs AG + GG	p = 0.028; OR = 1.28; 95% CI: 1.03-1.60
	**rs991804**	CC vs CT + TT	p = 0.493; OR = 1.17; 95% CI: 0.75-1.82
**TIRAP**	**rs11220437**	TT vs TC + CC	p = 0.155; OR = 1.21; 95% CI: 0.93-1.58
	**rs591163**	GG + GA vs AA	p = 0.237; OR = 1.01; 95% CI: 0.79-1.30
	**rs8177352**	AA vs AG + GG	p = 0.913; OR = 2.06; 95% CI: 0.45-9.55
	**rs8177375**	AA vs AG + GG	p = 0.203; OR = 1.21; 95% CI: 0.90-1.61
	**rs8177376**	AA vs AC + CC	p = 0.004; OR = 1.42; 95% CI: 1.12-1.80
	**rs17866704**	TT vs TC + CC	p = 0.023; OR = 1.31; 95% CI: 1.04-1.66

**Table 2:** Association studies between CCC with a left ventricular ejection fraction value under 0.4% and ASY including as covariates the gender and the polymorphism one by one

**GENE**	**Tag SNP**	**Genotype groups**	**Association test**
**CCR5**	**rs3176763**	CC vs CA + AA	p = 0.005; OR = 1.88; 95% CI: 1.20-2.94
	**rs3087253**	AA vs AT + TT	p = 0.861; OR = 1.02; 95% CI: 0.80-1.31
	**rs11575815**	AA + AT vs TT	p = 0.138; OR = 1.29; 95% CI: 0.92-1.82
**CCL2**	**rs3760396**	GG vs GA + AA	p = 0.920; OR = 1.02; 95% CI: 0.77-1.35
	**rs2857656**	CC vs CG + GG	p = 0.514; OR = 1.08; 95% CI: 0.85-1.39
	**rs4586**	TT vs TC + CC	p = 0.034; OR = 1.34; 95% CI: 1.02-1.75
	**rs3917891**	CC vs CT + TT	p = 0.053; OR = 1.55; 95% CI: 1.00-2.41
	**rs2530797**	AA vs AG + GG	p = 0.005; OR = 1.42; 95% CI: 1.11-1.82
	**rs991804**	CC vs CT + TT	p = 0.824; OR = 1.06; 95% CI: 0.65-1.73
**TIRAP**	**rs11220437**	TT vs TC + CC	p = 0.181; OR = 1.22; 95% CI: 0.91-1.63
	**rs591163**	GG + GA vs AA	p = 0.188; OR = 1.39; 95% CI: 0.88-1.90
	**rs8177352**	AA vs AG + GG	p = 0.858; OR = 1.02; 95% CI: 0.78-1.34
	**rs8177375**	AA vs AG + GG	p = 0.174; OR = 1.25; 95% CI: 0.91-1.69
	**rs8177376**	AA vs AC + CC	p = 0.005; OR = 1.46; 95% CI: 1.12-1.91
	**rs17866704**	TT vs TC + CC	p = 0.087; OR = 1.25; 95% CI: 0.97-1.62

**Table 3:** Association studies performed on an independent cohort including as covariates the gender and the polymorphism one by one

**CCC VS ASY**			
**GENE**	**Tag SNP**	**Genotype groups**	**Association test**
**CCL2**	**rs3760396**	GG vs GA + AA	p = 0.626; OR = 1.35; 95% CI: 0.40-4.55
	**rs2857656**	CC vs CG + GG	p = 0.267; OR = 1.16; 95% CI: 0.89-1.51
	**rs4586**	TT vs TC + CC	p = 0.128; OR = 1.25; 95% CI: 1.94-1.67
	**rs3917891**	CC vs CT + TT	p = 0.127; OR = 1.42; 95% CI: 0.90-2.23
	**rs2530797**	AA vs AG + GG	p = 0.007; OR = 1.46; 95% CI: 1.11-1.92
	**rs991804**	CC vs CT + TT	p = 0.435; OR = 1.23; 95% CI: 0.73-2.09
**TIRAP**	**rs11220437**	TT vs TC + CC	p = 0.149; OR = 1.27; 95% CI: 0.92-1.75
	**rs591163**	GG + GA vs AA	p = 0.154; OR = 1.32; 95% CI: 0.90-1.94
	**rs8177352**	AA vs AG + GG	p = 0.278; OR = 1.20; 95% CI: 0.87-1.66
	**rs8177375**	AA vs AG + GG	p = 0.256; OR = 1.22; 95% CI: 0.87-1.72
	**rs8177376**	AA vs AC + CC	p = 0.037; OR = 1.36; 95% CI: 1.19-1.80
	**rs17866704**	TT vs TC + CC	p = 0.051; OR = 1.32; 95% CI: 1.00-1.76
**CCC with a left ventricular ejection fraction value under 0.4% VS ASY**			
**GENE**	**Tag SNP**	**Genotype groups**	**Association test**
**CCL2**	**rs3760396**	GG vs GA + AA	p = 0.392; OR = 1.84; 95% CI: 0.45-7.46
	**rs2857656**	CC vs CG + GG	p = 0.499; OR = 1.10; 95% CI: 0.83-1.46
	**rs4586**	TT vs TC + CC	p = 0.194; OR = 1.23; 95% CI: 0.90-1.67
	**rs3917891**	CC vs CT + TT	p = 0.156; OR = 1.40; 95% CI: 0.88-2.24
	**rs2530797**	AA vs AG + GG	p = 0.002; OR = 1.59; 95% CI: 1.19-2.13
	**rs991804**	CC vs CT + TT	p = 0.876; OR = 1.05; 95% CI: 0.60-1.83
**TIRAP**	**rs11220437**	TT vs TC + CC	p = 0.265; OR = 1.21; 95% CI: 0.86-1.71
	**rs591163**	GG + GA vs AA	p = 0.134; OR = 1.38; 95% CI: 0.91-2.10
	**rs8177352**	AA vs AG + GG	p = 0.224; OR = 1.23; 95% CI: 0.88-1.73
	**rs8177375**	AA vs AG + GG	p = 0.313; OR = 1.21; 95% CI: 0.84-1.74
	**rs8177376**	AA vs AC + CC	p = 0.046; OR = 1.36; 95% CI: 1.05-1.85
	**rs17866704**	TT vs TC + CC	p = 0.095; OR = 1.29; 95% CI: 0.96-1.74

In multivariates analyses, several polymorphisms and gender were included as covariates. All the covariates are analyzed in the same time. In a stepwise approach, the worse associated covariate (non significant) is removed and the analysis is run again up to keep only significant associated covariates.

## Results and discussion

Fifteen Tag SNPs were genotyped successfully on our original cohort including ASY subjects (*n* = 118) and CCC patients (*n* = 315) (Table 4). The genotyping steps were done successfully for all the Tag SNPs. The genotype distribution of each SNP is summarized in Table 5. All the SNPs were in Hardy-Weinberg equilibrium on the ASY individuals considered as control subjects (p > 0,001) (Table 6).

**Table 4:** List of the tag SNPs genotyped on the original study population

**GENE**	**Tag SNP**	**Position relative to coordinate system**	**Position relative to transcription start point**
**CCR5**	rs3176763 C/A	46414281	−113
	rs3087253 A/G	46418689	+4295
	rs11575815 A/T	46420170	+5776
**CCL2**	rs3760396 G/C	32581441	−928
	rs2857656 C/G	32582007	−362
	rs4586 T/C	32583269	+900
	rs3917891 C/T	32585687	+3318
	rs2530797 A/G	32586094	+3725
	rs991804 C/T	32587725	+5356
**TIRAP**	rs11220437 T/C	126148160	−12630
	rs591163 G/A	126148432	−12358
	rs8177352 A/G	126153843	−6947
	rs8177375 A/G	126163064	+2274
	rs8177376 A/C	126163612	+2822
	rs17866704 T/C	126165757	+4967

**Table 5:** Genotype distribution on controls (ASY individuals) and cases (CCC) taking into account the gender and the left ventricular ejection fraction values

			**ASY**			**CCC**			**CCC (EF ≤ 0.4)**			**CCC (EF ≥ 0.4)**		
**Gene**	**SNP**	**Genotype**	** Total**	** Male**	**Female**	** Total**	** Male**	**Female**	** Total**	** Male**	**Female**	** Total**	** Male**	**Female**
**CCR5**	**rs3176763**	**CC**	**110**	50	59	**266**	167	97	**169**	127	42	**88**	34	54
			**(94.0%)**	(96.2%)	(92.2%)	**(84.4%)**	(87.0%)	(80.2%)	**(84.9%)**	(88.2%)	(76.4%)	**(89.0%)**	(81.0%)	(84.4%)
		**CA**	**7**	2	5	**48**	25	23	**30**	17	13	**17**	8	9
			**(6.0%)**	(3.8%)	(7.8%)	**(15.2%)**	(13.0%)	(19.0%)	**(15.1%)**	(11.8%)	(23.6%)	**(16.0%)**	(19.0%)	(14.1%)
		**AA**	**0**	0	0	**1**	0	1	**0**	0	0	**1**	0	1
			**(0.0%)**	(0.0%)	(0.0%)	**(0.3%)**	(0.0%)	(0.8%)	**(0.0%)**	(0.0%)	(0.0%)	**(0.9%)**	(0.0%)	(1.6%)
**CCR5**	**rs3087253**	**AA**	**46**	18	28	**134**	81	52	**80**	61	19	**52**	20	32
			**(41.4%)**	(36.7%)	(45.9%)	**(43.9%)**	(43.5%)	(44.4%)	**(41.0%)**	(43.3%)	(35.2%)	**(51.0%)**	(50.0%)	(51.6%)
		**AG**	**47**	22	24	**119**	73	46	**80**	52	28	**34**	16	18
			**(42.3%)**	(44.9%)	(39.3%)	**(39.0%)**	(39.2%)	(39.3%)	**(41.0%)**	(36.9%)	(51.9%)	**(33.3%)**	(40.0%)	(29.0%)
		**GG**	**18**	9	9	**52**	32	19	**35**	28	7	**16**	4	12
			**(16.2%)**	(18.4%)	(14.8%)	**(17.0%)**	(17.2%)	(16.2%)	**(17.9%)**	(19.9%)	(13.0%)	**(15.7%)**	(10.0%)	(19.4%)
**CCR5**	**rs11575815**	**AA**	**51**	25	25	**158**	97	60	**104**	74	22	**51**	22	29
			**(45.1%)**	(49.0%)	(41.0%)	**(51.3%)**	(51.6%)	(50.8%)	**(52.8%)**	(52.1%)	(53.7%)	**(50.0%)**	(53.7%)	(47.5%)
		**AT**	**42**	20	22	**120**	70	49	**71**	50	16	**43**	16	27
			**(37.2%)**	(39.2%)	(36.1%)	**(39.0%)**	(37.2%)	(41.5%)	**(36.0%)**	(35.2%)	(39.0%)	**(42.2%)**	(39.0%)	(44.3%)
		**TT**	**20**	6	14	**30**	21	9	**22**	18	3	**8**	3	5
			**(17.7%)**	(11.8%)	(23.0%)	**(9.7%)**	(11.2%)	(7.6%)	**(11.2%)**	(12.7%)	(7.3%)	**(7.8%)**	(7.3%)	(8.2%)
**CCL2**	**rs3760396**	**GG**	**87**	41	45	**247**	152	94	**153**	112	41	**87**	36	51
			**(75.7%)**	(80.4%)	(71.4%)	**(79.9%)**	(80.9%)	(79.0%)	**(77.7%)**	(78.9%)	(74.5%)	**(85.3%)**	(90.0%)	(82.3%)
		**GC**	**27**	9	18	**60**	35	24	**43**	29	14	**14**	4	10
			**(23.5%)**	(17.6%)	(28.6%)	**(19.4%)**	(18.6%)	(20.2%)	**(21.8%)**	(20.4%)	(25.5%)	**(13.7%)**	(10.0%)	(16.1%)
		**CC**	**1**	1	0	**2**	1	1	**1**	1	0	**1**	0	1
			**(0.9%)**	(2.0%)	(0.0%)	**(0.6%)**	(0.5%)	(0.8%)	**(0.5%)**	(0.7%)	(0.0%)	**(1.0%)**	(0.0%)	(1.6%)
**CCL2**	**rs2857656**	**CC**	**50**	24	25	**122**	70	50	**77**	52	25	**42**	17	25
			**(44.2%)**	(48.0%)	(40.3%)	**(39.7%)**	(37.6%)	(42.0%)	**(39.3%)**	(36.9%)	(45.5%)	**(41.2%)**	(42.5%)	(40.3%)
		**CG**	**51**	22	29	**150**	92	58	**96**	72	24	**51**	18	33
			**(45.1%)**	(44.0%)	(46.8%)	**(48.9%)**	(49.5%)	(48.7%)	**(49.0%)**	(51.1%)	(43.6%)	**(50.0%)**	(45.0%)	(53.2%)
		**GG**	**12**	4	8	**35**	24	11	**23**	17	6	**9**	5	4
			**(10.6%)**	(8.0%)	(12.9%)	**(11.4%)**	(12.9%)	(9.2%)	**(11.7%)**	(12.1%)	(10.9%)	**(8.8%)**	(12.5%)	(6.5%)
**CCL2**	**rs4586**	**TT**	**38**	20	18	**74**	41	32	**46**	31	15	**26**	9	17
			**(34.5%)**	(40.8%)	(30.0%)	**(24.0%)**	(21.7%)	(27.4%)	**(23.4%)**	(21.8%)	(27.3%)	**(25.5%)**	(22.0%)	(27.9%)
		**TC**	**53**	23	29	**148**	90	57	**94**	70	24	**52**	19	33
			**(48.2%)**	(49.9%)	(48.3%)	**(48.1%)**	(47.6%)	(48.7%)	**(47.7%)**	(49.3%)	(43.6%)	**(51.0%)**	(46.3%)	(54.1%)
		**CC**	**19**	6	13	**86**	58	28	**57**	41	16	**26**	13	11
			**(17.3%)**	(12.2%)	(21.7%)	**(27.9%)**	(30.7%)	(23.9%)	**(28.9%)**	(28.9%)	(29.1%)	**(25.5%)**	(31.7%)	(18.0%)
**CCL2**	**rs3917891**	**CC**	**107**	47	59	**264**	161	101	**166**	120	46	**91**	37	54
			**(93.9%)**	(90.4%)	(96.7%)	**(86.0%)**	(86.1%)	(85.6%)	**(85.6%)**	(85.7%)	(85.2%)	**(88.3%)**	(90.2%)	(87.1%)
		**CT**	**7**	5	2	**41**	25	16	**26**	19	7	**12**	4	8
			**(6.1%)**	(9.6%)	(3.3%)	**(13.4%)**	(13.4%)	(13.6%)	**(13.4%)**	(13.6%)	(13.0%)	**(11.7%)**	(9.9%)	(12.9%)
		**TT**	**0**	0	0	**2**	1	1	**2**	1	1	**0**	0	0
			**(0.0%)**	(0.0%)	(0.0%)	**(0.7%)**	(0.5%)	(0.8%)	**(1.0%)**	(0.7%)	(1.9%)	**(0.0%)**	(0.0%)	(0.0%)
**CCL2**	**rs2530797**	**AA**	**47**	13	33	**163**	104	58	**110**	81	29	**47**	19	28
			**(41.6%)**	(25.5%)	(54.1%)	**(52.9%)**	(55.6%)	(48.7%)	**(55.8%)**	(57.0%)	(52.7%)	**(45.6%)**	(47.5%)	(44.4%)
		**AG**	**52**	33	19	**115**	68	46	**69**	51	18	**44**	16	28
			**(46.0%)**	(64.7%)	(31.1%)	**(37.3%)**	(36.4%)	(38.7%)	**(35%)**	(35.9%)	(32.7%)	**(42.7%)**	(40%)	(44.4%)
		**GG**	**14**	5	9	**30**	15	15	**18**	10	8	**12**	5	7
			**(12.4%)**	(9.8%)	(14.8%)	**(9.7%)**	(8.0%)	(12.6%)	**(9.1%)**	(7.0%)	(14.5%)	**(11.7%)**	(12.5%)	(11.1%)
**CCL2**	**rs991804**	**CC**	**51**	25	25	**120**	67	51	**78**	52	26	**40**	15	25
			**(45.5%)**	(48.1%	(42.4%)	**(41.2%)**	(38.3%)	(44.7%)	**(42.6%)**	(39.4%)	(51.0%)	**(40.4%)**	(39.5%)	(41.0%)
		**CT**	**53**	24	29	**148**	92	56	**88**	67	21	**54**	20	34
			**(47.3%)**	(46.2%)	(49.2%)	**(50.9%)**	(52.6%)	(51.1%)	**(48.1%)**	(50.8%)	(41.2%)	**(54.5%)**	(52.6%)	(55.7%)
		**TT**	**8**	3	5	**23**	16	7	**17**	13	4	**5**	3	2
			**(7.1%)**	(5.8%)	(8.5%)	**(7.9%)**	(9.1%)	(6.1%)	**(9.3%)**	(9.8%)	(7.8%)	**(5.1%)**	(7.9%)	(3.3%)
**TIRAP**	**rs11220437**	**TT**	**92**	44	47	**229**	142	85	**146**	106	40	**74**	31	43
			**(80.0%)**	(84.6%)	(75.8%)	**(73.9%)**	(74.7%)	(72.0%)	**(73.7%)**	(73.6%)	(74.1%)	**(71.8%)**	(75.6%)	(69.4%)
		**TC**	**22**	8	14	**76**	45	31	**48**	36	12	**28**	9	19
			**(19.1%)**	(15.4%)	(22.6%)	**(24.5%)**	(23.7%)	(26.3%)	**(24.2%)**	(25.0%)	(22.2%)	**(27.2%)**	(22.0%)	(30.6%)
		**CC**	**1**	0	1	**5**	3	2	**4**	2	2	**1**	1	0
			**(9.0%)**	(0.0%)	(1.6%)	**(1.6%)**	(1.6%)	(1.7%)	**(2.0%)**	(1.4%)	(3.7%)	**(1.0%)**	(2.4%)	(0.0%)
**TIRAP**	**rs591163**	**GG**	**51**	23	28	**158**	95	82	**104**	74	20	**52**	30	32
			**(46.4%)**	(46.9%)	(46.7%)	**(51.5%)**	(50.5%)	(53.0%)	**(53.1%)**	(52.1%)	(50.0%)	**(51.5%)**	(55.6%)	(52.5%)
		**GA**	**44**	19	24	**120**	76	43	**75**	56	16	**38**	19	22
			**(40.0%)**	(38.8%)	(40.0%)	**(39.1%)**	(40.4%)	(36.8%)	**(38.3%)**	(39.4%)	(40.0%)	**(37.6%)**	(35.2%)	(36.1%)
		**AA**	**15**	7	8	**29**	17	12	**17**	12	4	**11**	5	7
			**(13.6%)**	(14.3%)	(13.3%)	**(9.4%)**	(9.0%)	(10.3%)	**(8.7%)**	(8.5%)	(10.0%)	**(10.9%)**	(9.3%)	(11.5%)
**TIRAP**	**rs8177352**	**AA**	**83**	38	44	**225**	132	91	**140**	98	42	**80**	31	49
			**(73.5%)**	(4.5%)	(72.1%)	**(73.3%)**	(70.6%)	(77.1%)	**(71.4%)**	(69.5%)	(76.4%)	**(77.7%)**	(75.6%)	(79.0%)
		**AG**	**28**	12	16	**73**	48	25	**47**	36	11	**23**	10	13
			**(24.8%)**	(23.5%)	(26.2%)	**(23.8%)**	(25.7%)	(21.2%)	**(24.0%)**	(25.5%)	(20.0%)	**(22.3%)**	(24.4%)	(21.0%)
		**GG**	**2**	60	1	**9**	7	2	**9**	7	2	**0**	0	0
			**(1.8%)**	(23.5%)	(1.6%)	**(2.9%)**	(3.7%)	(1.7%)	**(4.6%)**	(5.0%)	(3.6%)	**(0.0%)**	(0.0%)	(0.0%)
**TIRAP**	**rs8177375**	**AA**	**95**	45	49	**246**	153	91	**156**	115	41	**82**	34	48
			**(84.1%)**	(88.2%)	(80.3%)	**(79.4%)**	(81.4%)	(75.8%)	**(79.2%)**	(81.0%)	(74.5%)	**(78.8%)**	(82.9%)	(76.2%)
		**AG**	**16**	5	11	**60**	33	27	**39**	26	13	**20**	6	14
			**(14.2%)**	(9.8%)	(18.0%)	**(19.4%)**	(17.6%)	(22.5%)	**(19.8%)**	(18.3%)	(23.6%)	**(19.2%)**	(14.6%)	(22.2%)
		**GG**	**2**	1	1	**4**	2	2	**2**	1	1	**2**	1	1
			**(1.8%)**	(2.0%)	(1.6%)	**(1.2%)**	(1.0%)	(1.7%)	**(1.0%)**	(0.7%)	(1.8%)	**(1.9%)**	(2.4%)	(1.6%)
**TIRAP**	**rs8177376**	**AA**	**63**	26	37	**230**	143	85	**149**	110	39	**74**	29	45
			**(54.9%)**	(57.8%)	(60.7%)	**(75.4%)**	(77.7%)	(71.4%)	**(76.8%)**	(79.1%)	(70.9%)	**(71.8%)**	(72.5%)	(71.4%)
		**AC**	**40**	18	22	**70**	40	30	**44**	28	16	**25**	11	14
			**(37.7%)**	(40.0%)	(36.1%)	**(23.0%)**	(21.7%)	(25.2%)	**(22.7%)**	(20.1%)	(29.1%)	**(24.3%)**	(27.5%)	(22.2%)
		**CC**	**3**	1	2	**5**	1	4	**1**	1	0	**4**	0	4
			**(2.8%)**	(2.2%)	(3.3%)	**(1.6%)**	(0.5%)	(3.4%)	**(0.5%)**	(0.7%)	(0.0%)	**(3.9%)**	(0.0%)	(6.3%)
**TIRAP**	**rs17866704**	**TT**	**80**	35	44	**175**	103	71	**115**	80	35	**54**	20	34
			**(70.8%)**	(70.0%)	(71.0%)	**(57.4%)**	(55.4%)	(60.7%)	**(59.0%)**	(56.7%)	(64.8%)	**(53.5%)**	(50.0%)	(55.7%)
		**TC**	**32**	15	17	**106**	64	41	**63**	46	17	**40**	16	24
			**(28.3%)**	(30.0%)	(27.4%)	**(34.8%)**	(34.4%)	(35.0%)	**(32.3%)**	(32.6%)	(31.5%)	**(39.6%)**	(40%)	(39.3%)
		**CC**	**1**	0	1	**24**	19	5	**17**	15	2	**7**	4	3
			**(0.9%)**	(0.0%)	(1.6%)	**(7.9%)**	(10.2%)	(4.3%)	**(8.7%)**	(10.6%)	(3.7%)	**(6.9%)**	(10.0%)	(4.9%)

**Table 6:** Hardy-Weinberg equilibrium test

**GENE**	**Tag SNP**	**Chi2**	**p**
**CCR5**	rs3176763 C/A	0.111257738	0.9458
	rs3087253 A/G	1.014584489	0.6021
	rs11575815 A/T	0.111257738	0.9458
**CCL2**	rs3760396 G/C	0.491314613	0.7821
	rs2857656 C/G	0.035595421	0.9823
	rs4586 T/C	0.004981781	0.9975
	rs3917891 C/T	0.114371123	0.9444
	rs2530797 A/G	0.004293958	0.9978
	rs991804 C/T	1.35646743	0.5075
**TIRAP**	rs11220437 T/C	0.063307752	0.9688
	rs591163 G/A	1.190573349	0.5514
	rs8177352 A/G	0.042221875	0.9791
	rs8177375 A/G	1.69100575	0.4293
	rs8177376 A/C	1.294967649	0.5233
	rs17866704 T/C	1.314206075	0.5183

### Polymorphisms rs3176763C/A and rs11575815A/T, around the CCR5 gene, are associated to an increased risk of CCC

Three tag SNPs were genotyped for the CCR5 gene. In the CCC subjects group, 266 (84.4%) subjects carried the rs3176763C/C genotype whereas 110 (94.0%) of the ASY controls carried this genotype. This difference was significant in an univariate analysis including also the gender as covariate (p = 0.006; OR = 1.79; 95% CI: 1.18-2.70) (see Table 1).

For the rs11575815A/T polymorphism, 278 (90.3%) CCC subjects carried the genotypes rs11575815A/A or rs11575815A/T versus 93 (82.3%) for the ASY controls. This difference was significant (p = 0.030; OR = 1.41; 95% CI: 1.03-1.92) (see Table 1).

We performed a multivariate analysis (binary regression, stepwise procedure) to confirm the associations found previously in univariate analysis. Similarly to the univariate analysis, the genotypes were considered as binary variables. In this analysis, we included rs3176763C/A, rs11575815A/T and the gender as covariates. Polymorphism rs3176763C/A (p = 0.014; OR = 1.69; 95% CI: 1.11-2.57) and the gender (p = 0.002; OR = 2.04; 95% CI: 1.31-3.19) were still significantly associated to CCC (see Table 7). A trend of association was detected for rs11575815A/T (p = 0.077; OR = 1.33; 95% CI: 0.97-1.82).

**Table 7:** Multivariate stepwise binary logistic regression analysis between CCC and ASY including as covariates the gender and the polymorphisms associated in univariate analysis gene by gene

**GENE:**	**CCR5**		
**Step**	**Covariates**	**Groups**	**Association test**
Step1	gender	Male vs Female	p = 0.002; OR = 2.042; 95% CI: 1.31-3.19
	rs3176763	CC vs CA + AA	p = 0.014; OR = 1.689; 95% CI: 1.11-2.57
	rs11575815	AA + AT vs TT	p = 0.077; OR = 1.328; 95% CI: 1.03-1.82
Step2	gender	Male vs Female	p = 0.001; OR = 2.058; 95% CI: 1.32-3.21
	rs3176763	CC vs CA + AA	p = 0.007; OR = 1.766; 95% CI: 1.16-2.68
	rs11575815	AA + AT vs TT	Excluded
**GENE:**	**CCL2**		
**Step**	**Covariates**	**Groups**	**Association test**
Step1	gender	Male vs Female	p = 0.002; OR = 2.056; 95% CI: 1.31-3.23
	rs2530797	AA vs AG + GG	p = 0.162; OR = 1.198; 95% CI: 1.07-1.54
	rs4586	TT vs TC + CC	p = 0.348; OR = 1.138; 95% CI: 1.15-1.49
	rs3917891	CC vs CT + TT	p = 0.131; OR = 1.392; 95% CI: 1.1-2.140
Step2	gender	Male vs Female	p = 0.002; OR = 2.070; 95% CI: 1.32-3.25
	rs2530797	AA vs AG + GG	p = 0.051; OR = 1.258; 95% CI: 1.00-1.59
	rs3917891	CC vs CT + TT	p = 0.095; OR = 1.435; 95% CI: 1.06-2.19
	rs4586	TT vs TC + CC	Excluded
Step3	gender	Male vs Female	p = 0.001; OR = 2.091; 95% CI: 1.33-3.28
	rs2530797	AA vs AG + GG	p = 0.022; OR = 1.303; 95% CI: 1.04-1.64
	rs4586	TT vs TC + CC	Excluded
	rs3917891	CC vs CT + TT	Excluded
**GENE:**	**TIRAP**		
**Step**	**Covariates**	**Groups**	**Association test**
Step1	gender	Male vs Female	p = 0.002; OR = 2.062; 95% CI: 1.30-3.27
	rs8177376	AA vs AC + CC	p = 0.013; OR = 1.357; 95% CI: 1.06-1.73
	rs17866704	TT vs TC + CC	p = 0.039; OR = 1.298; 95% CI: 1.01-1.66

When we compared the ASY subjects to severe CCC patients (left ventricular ejection fraction value under 0.4%), only the association of rs3176763C/A was maintained in univariate analysis (p = 0.005; OR = 1.88; 95% CI: 1.20-2.94) (see Table 2). These two markers (rs3176763C/A and rs11575815A/T) did not discriminate moderate CCC from severe CCC (p > 0.5).

### Polymorphisms rs4586T/C, rs3917891C/T and rs2530797A/G, around the CCL2 gene, are associated to an increased risk of CCC

Six tag SNPs were genotyped for the CCL2 gene. In the CCC subjects group, 74 (24.0%) carried the rs4586T/T genotype whereas 38 (34.5%) of the ASY controls carried this genotype. This difference was significant in an univariate analysis (p = 0.032; OR = 1.30; 95% CI: 1.02-1.65) (see Table 1).

For the rs3917891C/T polymorphism, 264 (86.0%) CCC subjects carried the rs3917891C/C genotype versus 107 (93.9%) for the ASY controls. This difference was significant (p = 0.037; OR = 1.56; 95% CI: 1.03-2.37) (see Table 1).

For the rs2530797A/G polymorphism, 163 (52.9%) CCC subjects carried the rs2530797A/A genotype versus 47 (41.6%) for the ASY controls. This difference was significant (p = 0.028; OR = 1.28; 95% CI: 1.03-1.60) (see Table 1).

The same polymorphisms were associated when we compared the ASY subjects to severe CCC patients (rs4586T/C: p = 0.034; OR = 1.34; 95% CI: 1.02-1.75; rs3917891C/T: p = 0.053; OR = 1.55; 95% CI: 1.00-2.41; rs2530797A/G: p = 0.005; OR = 1.42; 95% CI: 1.11-1.82) (see Table 2).

We performed multivariate analysis including these three polymorphisms and the gender as covariates. When we compared the ASY subjects to CCC patients, only the polymorphism rs2530797A/G and the gender remained significantly associated (rs2530797A/G: p = 0.022; OR = 1.30; 95% CI: 1.04-1.64; gender: p = 0.001; OR = 2.09; 95% CI: 1.33-3.28) (see Table 7).

The same result was obtained, when we compared the ASY subjects to severe CCC patients (rs2530797A/G: p = 8.51×10^-7^; OR = 1.46; 95% CI: 1.13-1.88; gender: p = 0.004; OR = 3.59; 95% CI: 2.16-5.97). These three markers (rs4586T/C, rs3917891C/T and rs2530797A/G) did not discriminate moderate CCC from severe CCC (p > 0.16).

### Polymorphism rs8177376A/C, around the MAL/TIRAP gene, is associated to an an increased risk of CCC

Six tag SNPs were genotyped for the MALTIRAP gene. For the rs8177376A/C polymorphism, 230 (75.4%) CCC subjects carried the rs8177376A/A genotype versus 63 (54.9%) for the ASY controls. This difference was significant (p = 0.004; OR = 1.42; 95% CI: 1.12-1.80) (see Table 1). The same result was obtained when the analysis was restricted to severe CCC (p = 0.005; OR = 1.46; 95% CI: 1.12-1.91) (see Table 2).

A statistically significant difference was also detected for the rs17866704T/C polymorphism (p = 0.023; OR = 1.31; 95% CI: 1.04-1.66) (see Table 1). In our cohort, 175 ((57.4%) CCC subjects carried the rs17866704T/T genotype versus 80 (70.8%) for the ASY controls. The two SNPs remained associated in a multivariate analysis (see Table 7).

Some trend of association was detected for the rs17866704T/C polymorphism when we compared the ASY subjects to the severe CCC patients (p = 0.087; OR = 1.25; 95% CI: 0.97-1.62) (see Table 2). The rs8177376A/C marker did not discriminate moderate CCC from severe CCC (p > 0.57).

### The associations of the CCL2 and MAL/TIRAP genes were confirmed in a cohort from the original reports

The original data reporting association between the CCL2 and TIRAP genes were done by *Ramasawmy et al.*[41,42]. These studies were done on 169 patients with CCC and 76 *T. cruzi* infected ASY individuals. Our present study population is partially overlapping with the original one described by *Ramasawmy et al*. So, we repeated the analysis for these two genes on our cohort after removing the common subjects. This independent cohort includes 110/118 ASY subjects and 281/315 CCC patients. Of 281 patients with CCC, 192 had severe ventricular dysfunction and were classified as having severe CCC. The genotype distribution of the CCL2 and TIRAP Tag SNPs, on this independent cohort, is summarized in Table 8. In association studies, the gender was also included as covariates.

**Table 8:** Genotype distribution on our independent cohort which included 110 ASY controls and 281 cases (CCC) taking into account the gender and the left ventricular ejection fraction values

			**ASY**			**CCC**			**CCC (EF ≤ 0.4)**			**CCC (EF ≥ 0.4)**		
**Gene**	**SNP**	**Genotype**	** Total**	** Male**	**Female**	** Total**	** Male**	**Female**	** Total**	** Male**	**Female**	** Total**	** Male**	**Female**
**CCL2**	**rs3760396**	**GG**	**61**	30	30	**208**	128	79	**140**	99	41	**76**	28	36
			**(81.3%)**	(81.1%)	(81.1%)	**(80.0%)**	(80.9%)	(76.7%)	**(78.7%)**	(80.5%)	(74.5%)	**(86.4%)**	(93.3%)	(78.3%)
		**GC**	**13**	6	7	**50**	26	23	**37**	23	14	**11**	2	9
			**(17.3%)**	(16.2%)	(18.9%)	**(19.4%)**	(19.2%)	(22.3%)	**(20.8%)**	(18.7%)	(25.5%)	**(12.5%)**	(6.7%)	(19.6%)
		**CC**	**1**	1	0	**2**	1	1	**1**	1	0	**1**	0	1
			**(1.4%)**	(2.7%)	(0.0%)	**(0.6%)**	(0.9%)	(1.0%)	**(0.5%)**	(0.8%)	(0.0%)	**(1.1%)**	(0.0%)	(2.1%)
**CCL2**	**rs2857656**	**CC**	**34**	16	17	**101**	57	42	**71**	46	25	**28**	11	17
			**(46.6%)**	(44,4%)	(47,2%)	**(38.7%)**	(36.8%)	(42.0%)	**(39.9%)**	(37,4%)	(45.5%)	**(36,4%)**	(36,7%)	(36,2%)
		**CG**	**36**	20	16	**133**	59	54	**86**	62	24	**45**	16	29
			**(49.3%)**	(55,6%)	(44,4%)	**(51%)**	(51%)	(51,9%)	**(48,3%)**	(50,4%)	(43.6%)	**(58,4%)**	(53,3%)	(61,7%)
		**GG**	**3**	0	3	**27**	19	8	**21**	15	6	**4**	3	1
			**(4,1%)**	(0%)	(8,3%)	**(10.3%)**	(12.3%)	(7,7%)	**(11.8%)**	(12.2%)	(10.9%)	**(5,2%)**	(10%)	(2,1%)
**CCL2**	**rs4586**	**TT**	**23**	13	10	**62**	34	27	**44**	29	15	**17**	5	12
			**(32,4%)**	(37,1%)	(28,6%)	**(23,8%)**	(21.8%)	(26,5%)	**(24,6%)**	(23,4%)	(27.3%)	**(22,4%)**	(16,7%)	(26,1%)
		**TC**	**39**	20	18	**130**	78	51	**84**	60	24	**44**	17	27
			**(54,9%)**	(57,1%)	(51,4%)	**(50%)**	(50%)	(50%)	**(46,9%)**	(48,4%)	(43.6%)	**(57,9%)**	(56,7%)	(58,7%)
		**CC**	**9**	2	7	**68**	44	24	**51**	35	16	**15**	8	7
			**(12,7%)**	(5,7%)	(20%)	**(22.2%)**	(28,2%)	(23.5%)	**(28.5%)**	(28.2%)	(29.1%)	**(19,7%)**	(26,7%)	(15,2%)
**CCL2**	**rs3917891**	**CC**	**69**	34	34	**220**	132	86	**150**	104	46	**66**	27	39
			**(92%)**	(89,5%)	(94,4%)	**(84.9%)**	(85,2%)	(84,3%)	**(84,7%)**	(84,6%)	(85.2%)	**(86,8%)**	(90%)	(84,8%)
		**CT**	**6**	4	2	**37**	22	15	**25**	18	7	**10**	3	7
			**(8%)**	(10,5%)	(5,6%)	**(14,3%)**	(14,2%)	(14,7%)	**(14,1%)**	(14.6%)	(13.0%)	**(13,2%)**	(10%)	(15,2%)
		**TT**	**0**	0	0	**2**	1	1	**2**	1	1	**0**	0	0
			**(0%)**	(0%)	(0.0%)	**(0.8%)**	(0.6%)	(1%)	**(1.1%)**	(0.8%)	(1.9%)	**(0.0%)**	(0.0%)	(0.0%)
**CCL2**	**rs2530797**	**AA**	**25**	7	17	**132**	81	50	**96**	67	29	**32**	12	20
			**(33.8%)**	(18.9%)	(47.2%)	**(50.6%)**	(51.9%)	(48.5%)	**(53.6%)**	(54.0%)	(52,7%)	**(41.6%)**	(40.0%)	(42.6%)
		**AG**	**40**	26	14	**104**	62	41	**65**	47	18	**38**	15	23
			**(54.1%)**	(70.3%)	(38.9%)	**(39.8%)**	(39.7%)	(39.8%)	**(36.3%)**	(37.9%)	(32.7%)	**(49.4%)**	(50%)	(48.9%)
		**GG**	**9**	4	5	**25**	13	12	**18**	10	8	**7**	3	4
			**(12.2%)**	(10.8%)	(13.9%)	**(9.6%)**	(8.3%)	(11.7%)	**(10.1%)**	(8.1%)	(14,5%)	**(9.1%)**	(10.0%)	(8.5%)
**CCL2**	**rs991804**	**CC**	**35**	16	18	**99**	55	42	**71**	45	26	**26**	10	16
			**(47.6%)**	(42,1%)	(50%)	**(40,9%)**	(38.7%)	(42,9%)	**(43%)**	(39.5%)	(51.0%)	**(36,1%)**	(37%)	(35,6%)
		**CT**	**36**	21	15	**122**	72	50	**77**	56	21	**43**	15	28
			**(48%)**	(55,3%)	(41,7%)	**(50.4%)**	(50,7%)	(51%)	**(46,7%)**	(49,1%)	(41.2%)	**(59,7%)**	(55.6%)	(62,2%)
		**TT**	**4**	1	3	**21**	15	6	**17**	13	4	**3**	2	1
			**(5,3%)**	(2,6%)	(8.3%)	**(8,7%)**	(10,6%)	(6.1%)	**(10.3%)**	(11,4%)	(7.8%)	**(4,2%)**	(7.4%)	(2,2%)
**TIRAP**	**rs11220437**	**TT**	**61**	30	30	**189**	114	73	**132**	92	40	**51**	20	31
			**(81,3%)**	(79,8%)	(83,3%)	**(73.0%)**	(73,5%)	(71,6%)	**(74,2%)**	(74,2%)	(74.1%)	**(68%)**	(69%)	(67,4%)
		**TC**	**13**	8	5	**65**	38	27	**42**	30	12	**23**	8	15
			**(17,3%)**	(21,1%)	(13,9%)	**(25,1%)**	(24,5%)	(26.5%)	**(23,6%)**	(24,2%)	(22.2%)	**(30,7%)**	(27,6%)	(32,6%)
		**CC**	**1**	0	1	**5**	3	2	**4**	2	2	**1**	1	0
			**(1,3%)**	(0.0%)	(2,8%)	**(1.9%)**	(1.9%)	(2%)	**(2.2%)**	(1.6%)	(3.7%)	**(1.3%)**	(3.4%)	(0.0%)
**TIRAP**	**rs591163**	**GG**	**27**	14	13	**132**	77	54	**91**	61	30	**40**	16	24
			**(38%)**	(40%)	(37,1%)	**(50,8%)**	(49,7%)	(52,4%)	**(51,4%)**	(49,6%)	(55,6%)	**(51.9%)**	(53,3%)	(51,1%)
		**GA**	**33**	15	17	**103**	63	39	**70**	51	19	**29**	11	18
			**(46,5%)**	(42,9%)	(48,6%)	**(39.6%)**	(40,6%)	(37,9%)	**(39,5%)**	(41,5%)	(35,2%)	**(37.7%)**	(36,7%)	(38,3%)
		**AA**	**11**	6	5	**25**	15	10	**16**	11	5	**8**	3	5
			**(15,5%)**	(17,1%)	(14,3%)	**(9.6%)**	(9.7%)	(9,7%)	**(9%)**	(8.9%)	(9,3%)	**(10.4%)**	(10%)	(10,6%)
**TIRAP**	**rs8177352**	**AA**	**59**	30	28	**192**	111	79	**129**	87	42	**59**	22	37
			**(80,8%)**	(81,1%)	(80%)	**(74,1%)**	(71.6%)	(77.5%)	**(72,5%)**	(70,7%)	(76.4%)	**(77.6%)**	(73,3%)	(80,4%)
		**AG**	**13**	6	7	**58**	37	21	**40**	29	11	**17**	8	9
			**(17.8%)**	(16,2%)	(20%)	**(22,4%)**	(23,9%)	(20,6%)	**(22,5%)**	(23,6%)	(20.0%)	**(22.4%)**	(26,7%)	(19,6%)
		**GG**	**1**	1	0	**9**	7	2	**9**	7	2	**0**	0	0
			**(1.4%)**	(2,7%)	(0%)	**(3,5%)**	(4,5%)	(2%)	**(5,1%)**	(5.7%)	(3.6%)	**(0.0%)**	(0.0%)	(0.0%)
**TIRAP**	**rs8177375**	**AA**	**61**	32	28	**203**	124	77	**140**	99	41	**58**	24	34
			**(83,6%)**	(86,5%)	(80%)	**(77,8%)**	(80%)	(74%)	**(78,7%)**	(80,5%)	(74.5%)	**(75,3%)**	(80%)	(72,3%)
		**AG**	**10**	4	6	**54**	29	25	**36**	23	13	**17**	5	12
			**(13,7%)**	(10,8%)	(17,1%)	**(20,7%)**	(18,7%)	(24%)	**(20,2%)**	(18.7%)	(23.6%)	**(22,1%)**	(16,7%)	(25,5%)
		**GG**	**2**	1	1	**4**	2	2	**2**	1	1	**2**	1	1
			**(2,7%)**	(2,7%)	(2,9%)	**(1.5%)**	(1.3%)	(1.9%)	**(1.1%)**	(0.8%)	(1.8%)	**(2,6%)**	(3,3%)	(2,1%)
**TIRAP**	**rs8177376**	**AA**	**42**	19	23	**195**	121	72	**134**	95	39	**56**	24	32
			**(61,8%)**	(59,4%)	(63,9%)	**(75,6%)**	(78,6%)	(70,6%)	**(75,7%)**	(77,9%)	(70.9%)	**(73,3%)**	(80%)	(69,6%)
		**AC**	**25**	13	12	**60**	32	28	**42**	26	16	**18**	6	12
			**(36,8%)**	(40.6%)	(33,3%)	**(23.3%)**	(20,8%)	(27,5%)	**(23,7%)**	(21,3%)	(29.1%)	**(23,7%)**	(20%)	(26,1%)
		**CC**	**1**	0	1	**3**	1	2	**1**	1	0	**2**	0	2
			**(1,5%)**	(0%)	(2,8%)	**(1.2%)**	(0.6%)	(2%)	**(0.6%)**	(0.8%)	(0.0%)	**(2,6%)**	(0.0%)	(4.3%)
**TIRAP**	**rs17866704**	**TT**	**54**	27	26	**150**	85	71	**105**	70	35	**41**	14	27
			**(72%)**	(75%)	(68,4%)	**(58,4%)**	(55,2%)	(60.7%)	**(59,7%)**	(57,4%)	(64.8%)	**(54,7%)**	(46,7%)	(60%)
		**TC**	**20**	9	11	**88**	55	41	**59**	42	17	**27**	12	15
			**(26,7%)**	(25%)	(28,9%)	**(34,2%)**	(35,7%)	(35.0%)	**(33,5%)**	(34,4%)	(31.5%)	**(36%)**	(40%)	(33,3%)
		**CC**	**1**	0	1	**19**	14	5	**12**	10	2	**7**	4	3
			**(1,3%)**	(0.0%)	(2.6%)	**(7,4%)**	(9,1%)	(4.3%)	**(6,8%)**	(8,2%)	(3.7%)	**(9,3%)**	(13,3%)	(6,7%)

For the CCL2rs2530797A/G polymorphism, 132 (50.6%) CCC subjects carried the rs2530797A/A genotype versus 25 (33.8%) for the ASY controls (see Table 8). This difference was significant (p = 0.007; OR = 1.4 p = 0.007; OR = 1.46; 95% CI: 1.11-1.926) (see Table 8). The same polymorphism remained associated when we compared the ASY subjects to severe CCC patients (p = 0.002; OR = 1.59; 95% CI: 1.19-2.13) (see Table 3).

For the MAL/TIRAPrs8177376A/C polymorphism, 195 (75.6%) CCC subjects carried the rs8177376A/A genotype versus 42 (61.8%) for the ASY controls (see Table 8). This difference was significant on the whole independent cohort (p = 0.037; OR = 1.36; 95% CI: 1.19-1.80) (see Table 3). The same result was obtained when the analysis was restricted to severe CCC (p = 0.046; OR = 1.36; 95% CI: 1.05-1.85) (see Table 3).

A trend of association was detected for the rs17866704T/C polymorphism in both analyses (p = 0.051; OR = 1.32; 95% CI: 1.00-1.76) (see Table 3) and (p = 0.095; OR = 1.29; 95% CI: 0.96-1.74) (see Table 3).

In order to detect interaction between the candidate genes a multivariate stepwise binary logistic regression analysis was performed on ASY subjects and CCC patients (see Table 9). In this analysis, we included the gender, rs11575815A/T, rs2530797A/G**,** rs8177376A/C and rs17866704T/C as covariates. Polymorphisms CCR5rs3176763C/A (p = 0.007; OR = 1.879; 95% CI: 1.19-1.89), TIRAP rs8177376A/C (p = 0.007; OR = 1.393; 95% CI: 1.09-1.77) and the gender (p = 0.001; OR = 2.226; 95% CI: 1.39-3.55) were still significantly associated to CCC (see Table 9). However, if we want to add a significant number of genes and polymorphisms at the first step of the multivariate analysis, the study population (which is one of the largest described so far) is underpowered. So, we are working toward obtaining a cohort between 1,500 and 2,000 subjects that would enable us to assess whether possessing a given combination of alleles in several SNPs contribute more strongly for prognosis than the individual SNPs.

**Table 9:** Multivariate stepwise binary logistic regression analysis between CCC and ASY including as covariates the gender and the polymorphisms associated in all the previous multivariate analysis

**Step**	**Covariates**	**Groups**	**Association test**
**Step1**	Gender	Male vs Female	p = 0.001; OR = 2.179; 95% CI: 1.36-3.49
	CCR5rs3176763	CC vs CA + AA	p = 0.014; OR = 1.763; 95% CI: 1.12-2.77
	TIRAP rs8177376	AA vs AC + CC	p = 0.014; OR = 1.363; 95% CI: 1.07-1.74
	TIRAP rs17866704	TT vs TC + CC	p = 0.048; OR = 1.291; 95% CI: 1.01-1.66
	CCL2rs2530797	AA vs AG + GG	p = 0.114; OR = 1.212; 95% CI: 1.04-1.54
	TIRAP rs8177376	AA vs AC + CC	p = 0.014; OR = 1.363; 95% CI: 1.07-1.74
**Step2**	Gender	Male vs Female	p = 0.001; OR = 2.179; 95% CI: 1.36-3.48
	CCR5rs3176763	CC vs CA + AA	p = 0.008; OR = 1.842; 95% CI: 1.17-2.88
	TIRAP rs8177376	AA vs AC + CC	p = 0.015; OR = 1.356; 95% CI: 1.06-1.73
	TIRAP rs17866704	TT vs TC + CC	p = 0.064; OR = 1.267; 95% CI: 1.01-1.63
	CCL2rs2530797	AA vs AG + GG	Excluded
**Step3**	Gender	Male vs Female	p = 0.001; OR = 2.226; 95% CI: 1.39-3.55
	CCR5rs3176763	CC vs CA + AA	p = 0.007; OR = 1.879; 95% CI: 1.19-1.89
	TIRAP rs8177376	AA vs AC + CC	p = 0.007; OR = 1.393; 95% CI: 1.09-1.77
	TIRAP rs17866704	TT vs TC + CC	Excluded
	CCL2rs2530797	AA vs AG + GG	Excluded

We conducted an association study on several previously studied candidate genes on a Brazilian population. Whereas previously studies were done on a limited number of subjects (CCC patients ranges from 27 to 169, ASY controls ranges from 27 to 132) our study was done on a main cohort including 433 Chagas disease patients from the states of Sao Paulo, Minas Gerais and Bahia states. These patients were classified as seropositive ASY (*n* = 118) or as having CCC (*n* = 315). Whereas, previous studies were done on a limited number of SNPs, here, a Tag SNPs approach was applied to catch all the genetic information from each candidate gene.

For the CCR5 gene, two markers were associated to CCC (rs3176763C/A and rs11575815A/T). The association of rs3176763C/A was confirmed in a multivariate analysis or in a univariate analysis focusing only on severe CCC cases. rs3176763C/A polymorphism is located in the promoter of the gene and may affect the binding of transcription factors. Although these SNPs were not studied before, is in line with the literature in studies performed in other Latin American countries with diverse ethnic compositions, where several SNPs were located in the 5′UTR of the CCR5 gene where they may influence binding of regulatory elements to gene expression control regions [22,47,48,61]. As suggested by Florez *et al.*, these polymorphisms do not act independently [61]. Multiple polymorphic changes in the promoter may influence in a differential way the levels of CCR5 expression and the type of cell in which it is expressed. So, it’s more appropriate to talk about a susceptibility haplotype rather than individual SNPs. The content and the length of this haplotype may vary from one population to the other. The subsets of patients that develop Chagas cardiomyopathy display an exacerbated Th1 immune response. The relevance of the CCR5 and CXCR3 chemokine–chemokine receptor axis in Th1 cell migration to the heart has been demonstrated in experimental models [62-64] and in CCC [22].

For the CCL2 gene, three markers were associated with CCC (rs4586T/C, rs3917891C/T and rs2530797A/G). Only the rs2530797A/G polymorphism remains associated into multivariate analysis. The rs4586C/T polymorphism is a synonymous marker, whereas the two other SNPs are located into the 3′ region of the gene and may affect stability of the transcript or the binding of regulatory elements. The previous associated marker reported by Ramasawmy *et al.* is located into the promoter region (CCL2-2518A-G known as rs1024611) [42]. These results are absolutely not in discrepancy. Indeed, our tag SNPs were selected on the CEU and YRI reference populations. In these two reference populations the rs2530797A/G and rs1024611 are in strong linkage disequilibrium (previous associated marker) (D’ = 1). So the genetic involvement of the CCL2 gene in the control of the human susceptibility to chronic disease is confirmed. Patients with severe Chagas disease had elevated plasma concentrations of TNF-α and CCL2. Moreover, there is a good correlation between levels of these proteins (especially TNF-α) and the degree of left ventricular dysfunction [14]. Real-time quantitative PCR analysis in human CCC myocardium showed that the gene expression levels of CCL2 was selectively upregulated [12], reinforcing the importance of regulation of CCL2 expression in the pathogenesis of CCC.

For the TIRAP gene, only one marker, located into the 3′UTR region of the gene, was strongly associated (rs8177376A/C) and may affect stability of the transcript or the binding of regulatory elements. This result is in line with previous association reported by Ramasawmy *et al.*[41] who reported a non-synonimous polymorphism at a coding region (TIRAP975C/T, S180L known also as rs8177374). Indeed these two SNPs (rs8177376 and rs8177374) are in strong linkage disequilibrium. This gene encodes for a TIR adaptor protein involved in the TLR4 signaling pathway of the immune system. It activates NF-kappa-B, MAPK1, MAPK3 and JNK, which promote cytokine secretion and the inflammatory response.

## Conclusions

Our data show beyond reasonable doubt that polymorphisms affecting key molecules involved in several immune parameters (innate immunity signal transduction and T cell/monocyte migration to inflammatory regions) play a role in genetic susceptibility to CCC development. However, the functional impact of these markers remains unknown. This also points out to the multigenic character of CCC, each polymorphism imparting a small contribution.

When all the genetic markers will be identified, we will be able to performed multivariate analyses using several genes (gene polymorphisms) as covariates. In order to perform this kind of analysis it is essential to enroll a study population including at least 1,500 and 2,000 cases and 1000 ASY controls. It will allow us to detect gene–gene interactions and additive or antagonist effects between the associated polymorphisms. A panel of markers will be defined to early detect individuals with a highest risk to develop chronic Chagas cardiomyopathy. It will provide information for pathogenesis as well as therapeutic targets. The identification of these marker sets may also have a combined prognostic value for disease progression at the individual patient level, allowing close follow up and early treatment of those carrying high-risk genetic signatures.

## Abbreviations

CCC: Chronic Chagas cardiomyopathy; ASY: Asymptomatic; Th1: T helper 1; SNP: Single nucleotide polymorphism; T. cruzi: *Trypanosoma cruzi*; PAMPs: Pathogen-associated molecular patterns; IL: Interleukin; TNF: Tumor necrosis factor; LVEF: Left ventricular ejection fraction.

## Competing interests

The authors declare that they have no competing interests.

## Authors’ contributions

Contribution to conception and design: JK, ACP, ECN, CC. Performed the experiments: AFF, MB. Analysis of the data: AFF PCT ECN CC. Contributed reagents materials analysis tools: CWP, BMI, BS, HTLW, LGN, ADMB, PB, FD, AS, ED, JAMN, MH, MS, AF, VR, ACP. Wrote the paper: ECN, CC. Review the drafts: ECN, CC, AFF, LRPF. All authors read and approved the final manuscript.

## Pre-publication history

The pre-publication history for this paper can be accessed here:

http://www.biomedcentral.com/1471-2334/13/587/prepub
